# RNA-seq analysis reveals transcriptome reprogramming and alternative splicing during early response to salt stress in tomato root

**DOI:** 10.3389/fpls.2024.1394223

**Published:** 2024-06-20

**Authors:** Jianghuang Gan, Yongqi Qiu, Yilin Tao, Laining Zhang, Thomas W. Okita, Yanyan Yan, Li Tian

**Affiliations:** ^1^ Collaborative Innovation Center for Efficient and Green Production of Agriculture in Mountainous Areas of Zhejiang Province, College of Horticulture Science, Zhejiang A&F University, Hangzhou, Zhejiang, China; ^2^ Key Laboratory of Quality and Safety Control for Subtropical Fruit and Vegetable, Ministry of Agriculture and Rural Affairs, College of Horticulture Science, Zhejiang A&F University, Hangzhou, Zhejiang, China; ^3^ Institute of Biological Chemistry, Washington State University, Pullman, WA, United States

**Keywords:** *Solanum lycopersicum*, tomato root, salt stress, alternative splicing, RNA sequencing, transcription factors

## Abstract

Salt stress is one of the dominant abiotic stress conditions that cause severe damage to plant growth and, in turn, limiting crop productivity. It is therefore crucial to understand the molecular mechanism underlying plant root responses to high salinity as such knowledge will aid in efforts to develop salt-tolerant crops. Alternative splicing (AS) of precursor RNA is one of the important RNA processing steps that regulate gene expression and proteome diversity, and, consequently, many physiological and biochemical processes in plants, including responses to abiotic stresses like salt stress. In the current study, we utilized high-throughput RNA-sequencing to analyze the changes in the transcriptome and characterize AS landscape during the early response of tomato root to salt stress. Under salt stress conditions, 10,588 genes were found to be differentially expressed, including those involved in hormone signaling transduction, amino acid metabolism, and cell cycle regulation. More than 700 transcription factors (TFs), including members of the MYB, bHLH, and WRKY families, potentially regulated tomato root response to salt stress. AS events were found to be greatly enhanced under salt stress, where exon skipping was the most prevalent event. There were 3709 genes identified as differentially alternatively spliced (DAS), the most prominent of which were serine/threonine protein kinase, pentatricopeptide repeat (PPR)-containing protein, E3 ubiquitin-protein ligase. More than 100 DEGs were implicated in splicing and spliceosome assembly, which may regulate salt-responsive AS events in tomato roots. This study uncovers the stimulation of AS during tomato root response to salt stress and provides a valuable resource of salt-responsive genes for future studies to improve tomato salt tolerance.

## Introduction

Soil salinization has become an increasingly serious global problem. It is estimated that more than 833 million hectares (8.7% of the Earth’s surface) are salinized worldwide with an annual increase of 10% (FAO, 2021). Soil salinization is projected to extend to more than 50% of the arable land by 2050 (Jamil et al., 2011). There are various reasons for soil salinization, including low rainfall, weathering of indigenous rocks, and inappropriate irrigation and fertilization during the cultivation process (Shrivastava and Kumar, 2015). Saline soils are known to suppress plant growth and development, which in turn severely affects crop yields in agricultural production (Yuan et al., 2016; van Zelm et al., 2020). Tomato (*Solanum lycopersicum* L.) is one of the most grown and valuable vegetable crops in the world, ranking the first among vegetable crops with an annual production of 186 million tons globally (FAO, 2022). Although tomato is thought to be moderately tolerant to salt stress, tomato yield and quality are severely affected by high salinity (Bonarota et al., 2022). The development of salt-tolerant tomato crops is therefore an important goal of plant breeding.

Salt stress can damage plant growth and development in many ways. High salt concentration in the soil modifies the structure of soil porosity and, in turn, hydraulic conductivity. This results in low water potential and nutrient availability, causing osmotic stress and eventually leading to metabolic toxicity and physiological disorders that affect plant growth and development (Tester and Davenport, 2003; Hasanuzzaman and Fujita, 2022; 2023). The rapid accumulation of reactive oxygen species (ROS) frequently occurs during salt stress, which induces oxidative stress, causes damage to cellular macromolecules like proteins and DNA, and destabilizes membranes and organelles (Kesawat et al., 2023). Furthermore, salt stress also decreases stomatal conductance and inhibits photosynthesis (Lawlor and Cornic, 2002; Chaves et al., 2009; Sayyad-Amin et al., 2016; Kesawat et al., 2023). All these negative effects impair most plant growth phases, from seed germination, vegetative growth, flowering and fruiting and eventually overall yield.

Along with the development of multi-omics technology, extensive studies have applied transcriptomics, proteomics, metabolomics or the combined analysis with biochemical and physiological characteristics to investigate the molecular mechanism underlying plant salt tolerance. Based on current understanding, plants adapt various mechanisms, including activation of osmotic adjustment, regulation of ion transport and homeostasis, clearance of reactive oxygen species, regulation of plant hormone signaling, modulation of cytoskeletal dynamics and the cell wall composition, to negate the adverse effects and survive at salinity condition (Wang et al., 2011; Hasanuzzaman and Fujita, 2022; Balasubramaniam et al., 2023). More importantly, regulation of gene expression is the integral part that activates and coordinates all these regulatory pathways.

Gene expression is regulated at transcriptional level mainly exerted by transcription factors and post-transcriptional events involving RNA processing, maturation, transport and turn-over (Zhao et al., 2017; Zhang et al., 2019). Alternative splicing (AS) is the main step during RNA processing to regulate gene expression and proteome diversity. As AS can generate multiple transcripts from a single RNA precursor via exon skipping, intron retention, and selection of alternative donor site or acceptor site as well as other intricate forms of splicing (Keren et al., 2010), AS eventually cause differential expression of the corresponding gene and modulate gene function via altering a protein domain or affecting the stability of the spliced transcript and the corresponding protein. Previous studies have demonstrated that high salinity stress can promote the occurrence of alternative splicing of stress-responsive genes and affect the expression of the genes coding spliceosome components in *Arabidopsis* (Ding et al., 2014b; Feng et al., 2015; Gu et al., 2018), rice (Yu et al., 2021; Jian et al., 2022), wheat (Liu et al., 2018), Barley (Fu et al., 2019), Date Palm (Xu et al., 2021), grapevine (Jin et al., 2021), cotton (Zhu et al., 2018), *Opisthopappus* (Han et al., 2024), etc. However, the alternative splicing events in tomato root under salt stress remains to be resolved.

In this study, we investigated the transcriptomic response of tomato root to salt stress, focusing on the global dynamics of transcriptome reprogramming and AS changes during the initial 12 hours under salt exposure. We found a large number of early response differentially expressed (DE) genes induced by salt stress while simultaneously elevating AS events of both DE and non-DE genes. Our findings provide a comprehensive understanding of tomato root response to salt stress and highlights the vital role of AS in tomato’s adaptation to salt stress.

## Results

### Overview of morphological performance of tomato seedlings and RNA-seq data of tomato roots in response to salt stress

**Figure 1:**
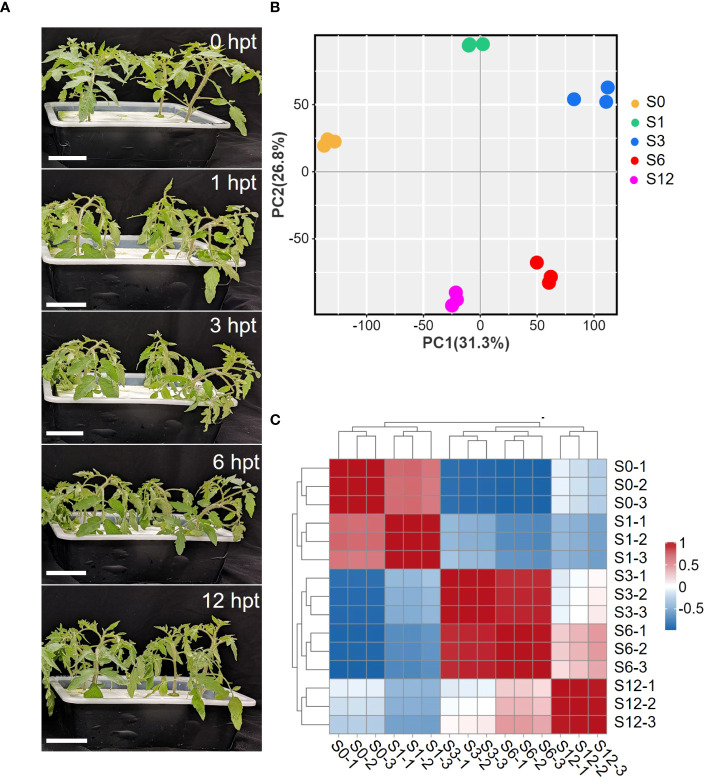
Phenotypic and transcriptome changes of tomato in response to salt stress. **(A)** Images of tomato plants at different treatment times (0, 1, 3, 6,12 hours) post treatment (hpt) in the presence of 150mM NaCl. **(B)** Principal component analysis (PCA) of RNA-seq data. Gene expression changes were investigated at 0h (S0), 1h (S1), 3h (S3), 6h (S6) and 12 hpt (S12) of salt stress treatment. The PCA was performed using normalized RNA-Seq data of all mapped genes. **(C)** Pearson’s correlation analysis of RNA-seq data between each sample.

To study the rapid response of tomato roots to salt stress, five-leaf-stage tomato seedlings were treated with 150 mM NaCl for 12 hours. At 1 hour post treatment (hpt), tomato leaves became dehydrated and wilted, exhibiting leaf curling and petiole softening. The dehydration of plants was more severe at 3 hpt but started to slightly recover at 6 hpt (**Figure 1A**). At 12 hpt, tomato plants apparently recovered from salt stress as plants showed upright growth without dehydration. The recovery beyond 6 hours suggests that tomato regulates changes in osmotic stress and restores ion homeostasis in a short amount of time after exposure to salt stress. In order to examine the underlying molecular mechanism of tomato’s early responses to salt stress, tomato roots were sampled at 0, 1, 3, 6 and 12 hpt (S0, S1, S3, S6, S12) and subjected to next generation RNA-sequencing. Three biological repeats per time point were performed and a total of 15 cDNA libraries were generated for sequencing. Approximately 6.9 billion raw reads were obtained and eventually around 6.6 billion high-quality reads (**Supplementary Tables S1**, **S2**) were mapped against the tomato genome to determine transcriptomic changes during early salt stress. Principal component analysis (PCA) (**Figure 1B**; **Supplementary Table S3**) and correlation analysis on RNA levels (**Figure 1C**; **Supplementary Table S4**) revealed excellent repeatability and reproducibility of the results. PCA showed an obvious separation of control group (S0) from salt treated groups, especially from the S3 and S6 samples (**Figure 1B**; **Supplementary Table S3**), suggesting that salt treatment significantly disturbed the transcriptome of tomato root.

### Transcriptional changes induced by salt stress in tomato root

**Figure 2:**
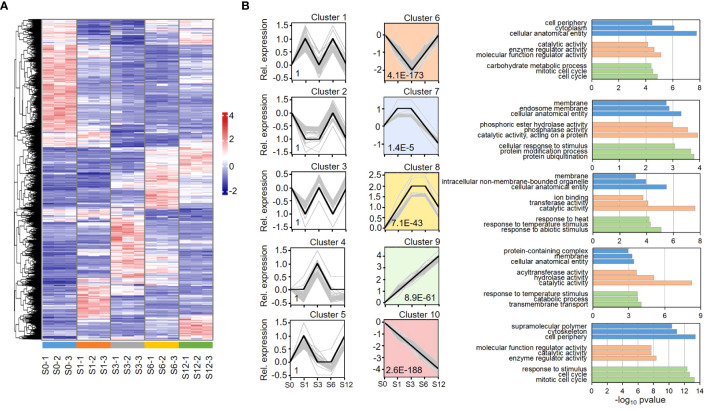
Hierarchical clustering and heatmap analyses of tomato DEGs. **(A)** Heatmap analysis shows dynamic expression pattern of DEGs during the early 12-hour response to salt stress in tomato root. **(B)** Hierarchical clustering analysis segregates DEGs into 10 clusters based on gene expression pattern. Y axis represents relative gene expression level based on normalized expression value. The *p* value of each cluster is shown inside the chart area, and the profound clusters are filled with colors. Top GO terms enriched in each profound cluster are shown on the right. GO terms of biological processes, molecular function and cellular component are shown in green, orange and blue bars. Detailed information of the clustered genes and list of all significant GO terms are provided in **Supplementary Tables S6** and **S7**, respectively.

Differential expression genes (DEGs) were firstly analyzed based on the value of FPKM (Fragments Per Kilobase of transcript per Million mapped reads). A gene was considered to be expressed if all three repeats showed FPKM > 0. DEGs were selected by a threshold of log2 fold change ≥ 1 and adjusted *p* value < 0.05 when compared to S0 group. Based on these criteria, total 10,588 DEGs out of 22,047 expressed genes were identified from the salt treated samples (**Supplementary Table S5**), indicating that nearly half of the expressed genes were impacted by salt stress. A heatmap analysis on the expression of all the DEGs revealed various expression patterns among the salt-impacted genes (**Figure 2A**). Based on their expression patterns, DEGs were classified into 10 clusters by hierarchical clustering analysis based on their expression pattern (**Figure 2B**; **Supplementary Tables S6**, **S7**). Among these clusters, the expression patterns in clusters 6 to 10 were significantly pronounced. The DEGs in cluster 6 were highly enriched in Gene Ontology (GO) terms of cellular anatomical entity, cytoplasm, cell periphery and mitotic cell cycles. These DEGs showed a decreased expression at the first 3 hours post salt treatment, suggesting that the process of cell differentiation was inhibited when tomato roots were exposed to salt stress. The DEGs in clusters 7 and 8 showed significantly increased expression in S1-S3 (Cluster 7) and S3-S6 (Cluster 8) samples, respectively. The DEGs in these clusters were highly enriched in membrane elements and the processes of stimulus response, reflecting a reconfiguration of the membrane under salt stress. Protein modification and ubiquitination were significantly enriched in cluster 7, revealing that protein turnover and metabolism was highly active during the first 3 hours of salt stress. The most pronounced expression patterns were observed in clusters 9 and 10 where DEGs showed linear enhancement (cluster 9) and depression (cluster 10) patterns along the treatment, respectively. The most highly enriched GO term in cluster 9 was catalytic activity, suggesting a continuous activation of enzymes during response to salt stress. Besides, GO terms of response to ROS and oxidative stress were also exclusively detected in cluster 9. Except in cluster 6, GO terms of mitotic cell cycle and cell periphery were also enriched in cluster 10. Multiple cell cycle related processes, such as cell cycle checkpoint signaling, DNA replication and phase transition, were enriched in cluster 6 and 10, further revealing a repression on cell differentiation during salt response.

Amino acid metabolic processes were significantly enriched in clusters 8 and 10. Cluster 8 contains genes involved in metabolism of aromatic amino acids, branched-chain amino acids, sulfur amino acids and alpha amino acids. While serine family amino acids catabolic process was pronounced in cluster 8, their biosynthetic process was only enriched in cluster 10. On the other hand, amino acid transmembrane transport was only significantly enriched in cluster 9. KEGG analysis (**Supplementary Figure S1**) further reveals that the metabolic pathways of many amino acids, including valine, leucine, isoleucine, serine, glycine, threonine, aspartate, glutamate, arginine, methionine, phenylalanine, tyrosine and tryptophan, were greatly influenced.

Biological processes of response to hormone were observed in highly enriched terms in clusters 6, 8 and 10, but not in cluster 9, suggesting that the process was highly dynamic but not continuously activated. Response to abscisic acid (ABA) and auxin were detected in both clusters 8 and 10. While response to cytokinin was enriched in cluster 10, responses to gibberellin and ethylene were enriched in cluster 8. KEGG analysis on plant hormone signaling pathways revealed that most of key steps in hormone signal transduction were significantly influenced in tomato root by salt stress (**Supplementary Figure S2**).

**Figure 3:**
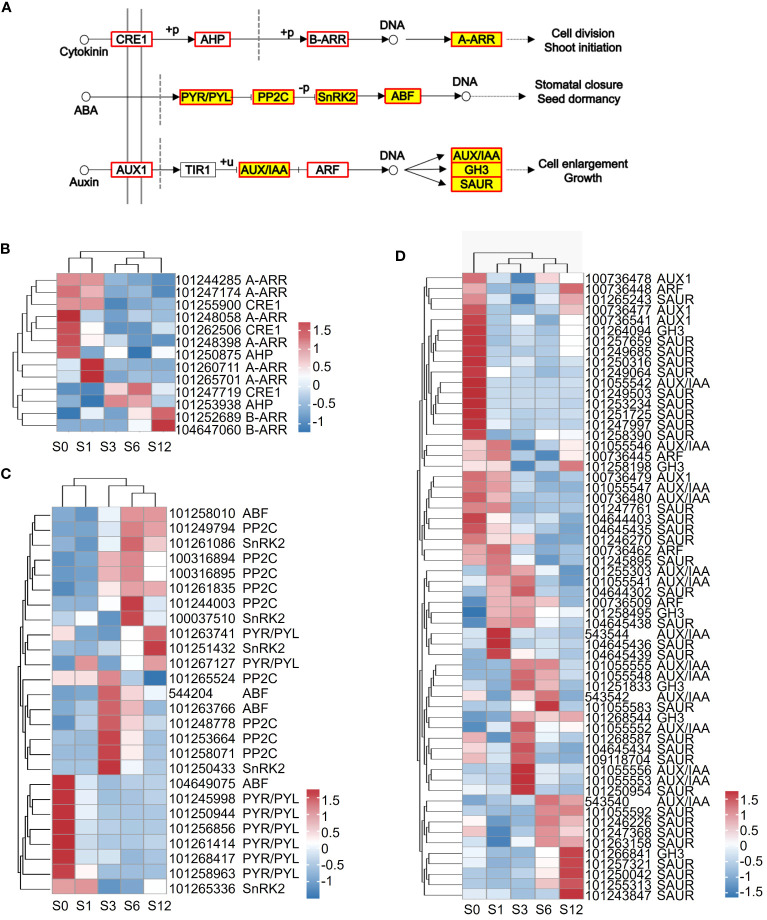
Expression of the annotated DEGs involved in plant hormone signal transduction KEGG pathway. **(A)** Overview of DEGs that code key factors functioning in cytokinin, ABA and auxin signal transduction. Red boxes represent genes that were regulated by salt stress, while red boxes filled in yellow represent the common genes found in the 1, 3, 6,12-hour samples treated under salt stress. **(B–D)** Heatmap analysis on the representative DEGs involved in cytokinin, ABA and auxin signaling transduction. The gene ID and potential family name are labeled on the right next to heatmap.

The genes involved in cytokinin, ABA and auxin signaling transduction showed various expression patterns as viewed by heatmap clustering (**Figure 3**; **Supplementary Table S9**), which reveals various expression pattern of these key factors. For example, while most of PYR/PYL genes was down-regulated by salt treatment, significant induction of PP2C was greatly observed at 3 hours after salt treatment (**Figure 3B**).

**Figure 4:**
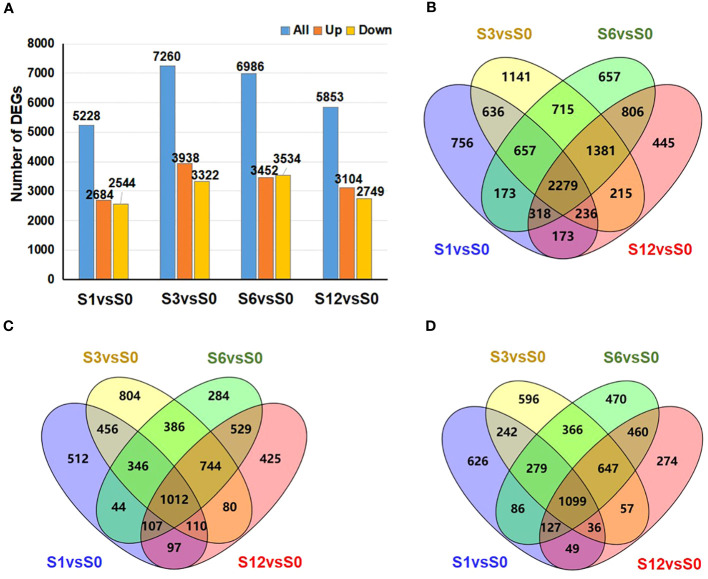
Number of DEGs during tomato root responses to salt stress. **(A)** Number of DEGs induced by salt treatment at different time point. Blue, orange and yellow bars represent the number of total DEGs, up-regulated DEGs, and down-regulated DEGs. **(B–D)** Venn diagram analysis to show the overlap or time-specific DEGs among different salt-treated groups. **(B)** all DEGs; **(C)** up-regulated DEGs; and **(D)** down-regulated DEGs.

**Figure 5:**
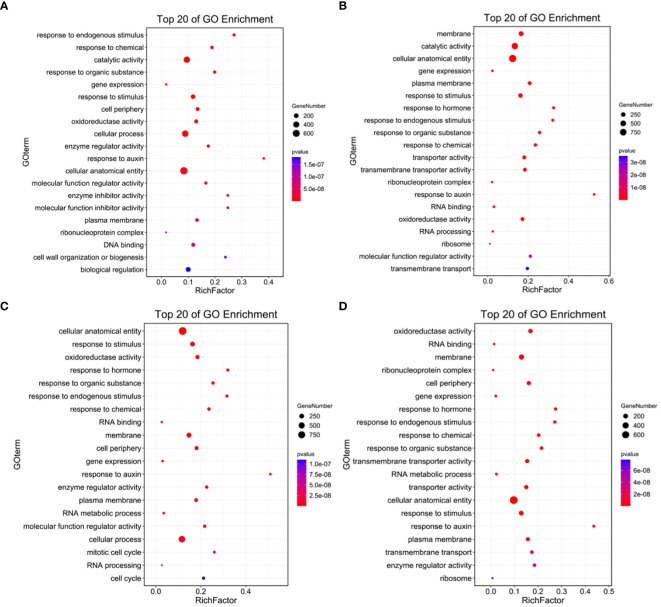
The enriched Gene Ontology (GO) terms of DEGs in salt treated samples. **(A–D)** Top 20 GO terms significantly enriched in the DEGs induced at 1 **(A)**, 3 **(B)**, 6 **(C)** and 12 **(D)** hours after salt treatment.

The numbers of DEGs (**Supplementary Table S8**) in the samples collected at each time point are shown in **Figure 4A**. The largest number of DEGs, 7,260 in total including 3,938 up-regulated and 3,322 down-regulated genes, was observed at 3 hours after salt treatment. Venn diagram data (**Figures 4B–D**) revealed 2,279 common DEGs (1,012 up-regulated and 1,099 down-regulated genes) among all pairwise comparisons (**Supplementary Table S10**). GO analyses were conducted to analyze the functions of all DEGs (**Figure 5**; **Supplementary Table S11**). Among the four comparison groups, several functional categories, including catalytic activity, cellular anatomical entity, cell periphery, membrane, response to hormone and response to stimulus, were strongly over-represented in all groups. It is worth noting that the DEGs from all salt treated samples were also highly enriched in the categories of RNA binding, RNA processing and RNA metabolic process (**Supplementary Table S11**), suggesting salt stress induces comprehensive changes in RNA metabolism. The common 2,279 DEGs contained genes are related to catalytic activity and responses to hormones and various stimuli (**Supplementary Figure S3**; **Supplementary Table S12**). Consistent with the previous clustering result, catalytic activity and cell cycle process were over-represented in the up-regulated and down-regulated common DEGs, respectively.

**Figure 6:**
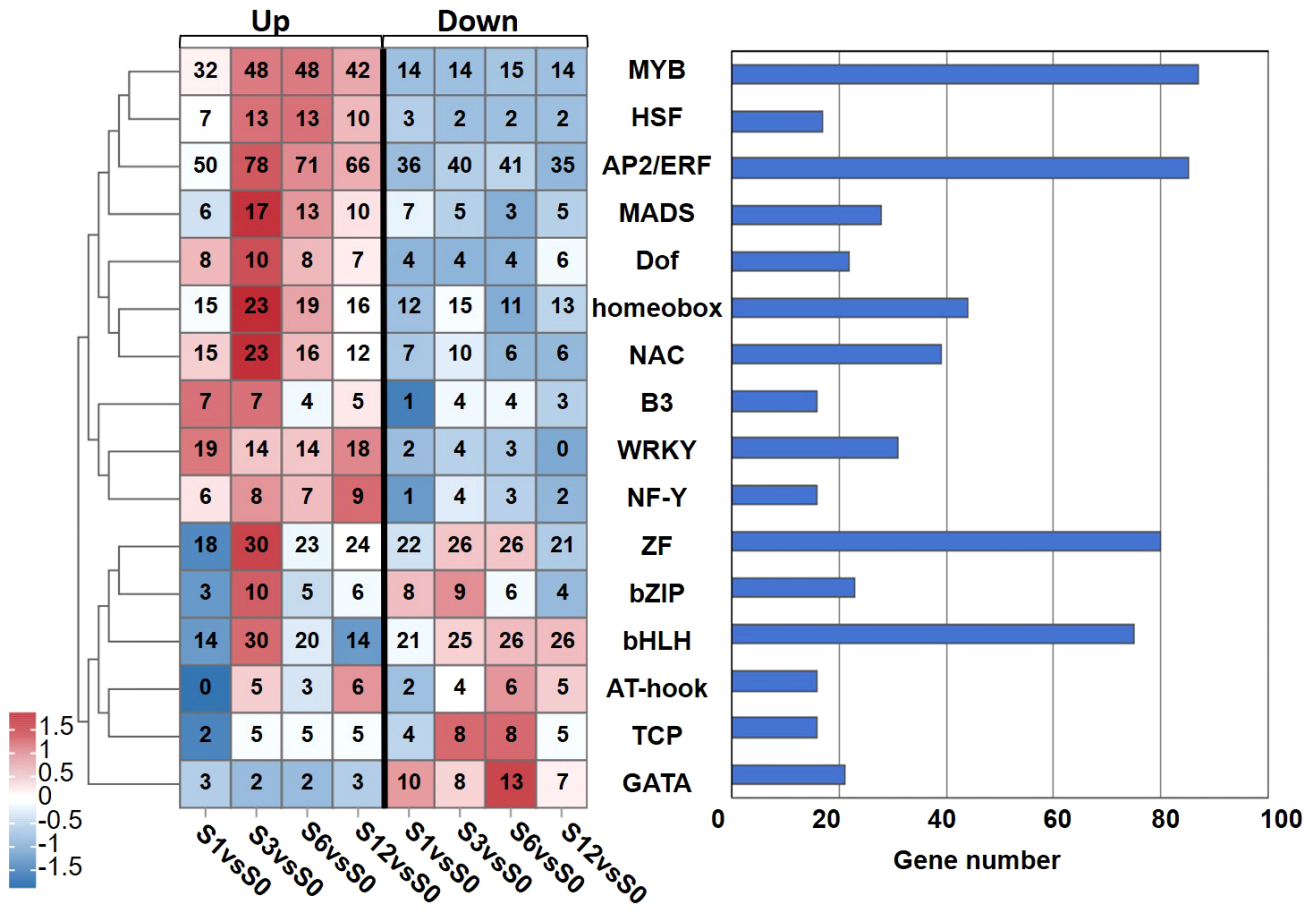
Numbers of the salt-responsive transcription factors (TFs) from the main TF families identified at different time points after salt treatment. Heatmap analysis on the distribution of up- and down-regulated TFs is shown in left panel, and total number of TFs from each family is presented in the chart on the right.

The expression of 738 transcription factors (TFs) belonging to 26 families were identified to be regulated by salt treatment (**Supplementary Table S13**). Among those TFs, 87 TFs were identified from the MYB family, 85 from the AP2/ethylene response factor (ERF) family, 80 from the zins finger (ZF) family, 75 from the bHLH family (**Figure 6**; **Supplementary Table S13**). Other TFs belonged to the superfamilies of homeobox (44), NAC (39), WRKY (31), MADS (28), bZIP (23), Dof (22) superfamilies were also noted. The distribution of these differentially expressed TFs along salt treatment is shown in **Figure 6**. A large proportion of TFs from MYB, heat stress transcription factor (HSF), AP2/ERF, MADS, Dof, homeobox, NAC, B3, WRKY and nuclear factor Y (NF-Y) families were up-regulated during the whole treatment. For example, 25 out of 31 WRKY TFs showed increased expression under salt stress (**Supplementary Table S13**). On the other hand, most of TFs from AT-hook, TCP, and GATA showed down-regulation. Members of ZF and bHLH TFs exhibited diverse expression patterns during salt treatment (**Figure 6**; **Supplementary Table S13**). Some of them were activated by salt stress while others were significantly down-regulated. We also noticed that TFs from MADS, homeobox and NAC families were highly induced at 3 hours after treatment.

### Analysis of alternative splicing events induced by salt stress

**Figure 7:**
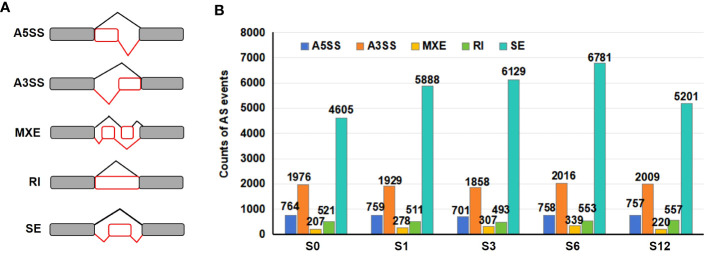
Distribution of alternative splicing events. **(A)** Diagram of five AS events detected in all 15 libraries. A5SS, alternative 5’splice site; A3SS, alternative 3’ splice site; MXE, mutually exclusive exon; RI, retained intron; and SE, skipped exon. **(B)** The number of each AS event in control and salt-treated groups.

Alternative splicing events were analyzed using the rMATS software based on transcript data. Five major types of AS patterns (**Figure 7A**), including alternative 5’ splice site (A5SS), alternative 3’ splice site (A3SS), mutually exclusive exon (MXE), retained intron (RI) and skipped exon (SE) were determined. A total of 11,217 A5SS, 29,363 A3SS, 4,052 MXE, 7,902 RI and 85,812 SE events were identified from all tested samples (**Supplementary Table S14**). The total numbers of AS events based on three repeats for each salt treated group (9,365 events for S1, 9,488 events for S3, 10,447 events for S6 and 8,743 events for S12 groups) were higher than that observed in S0 group (8,072) (**Figure 7B**; **Supplementary Table S14**), suggesting that salt stress promoted significant AS changes in the tomato root. SE event was the most common AS events, counting for 57% in S0, 62.9% in S1, 64.6% in S3, 64.9% in S6 and 59.5% in S12 group (**Supplementary Table S14**). A3SS was the second most abundant AS pattern (20.6–24.5%), followed by A5SS (7.3–9.5%), RI (5.2–6.5%) and MXE (2.5–3.2%) (**Supplementary Table S14**). Although the ratio of each AS type to the total AS events varied in individual group, the ratio of SE event was higher in salt treated samples than that in S0 sample.

**Figure 8:**
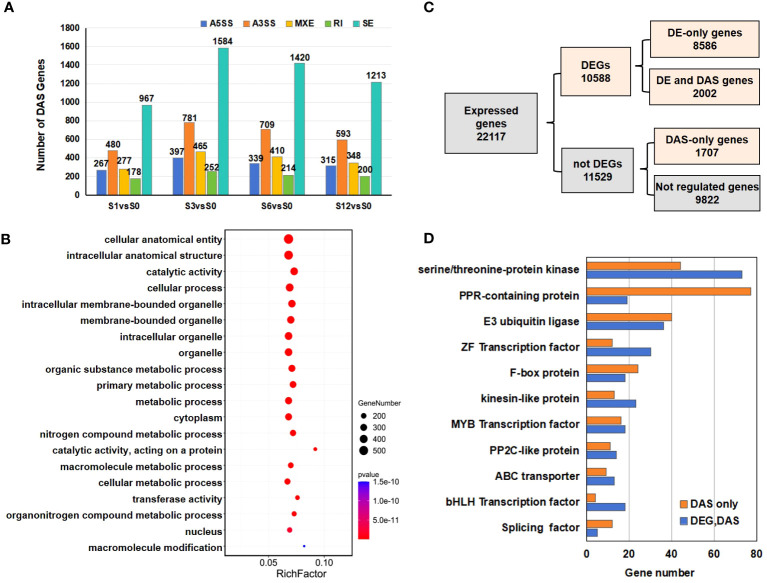
Numbers and functional analysis of DAS genes in salt treated samples. **(A)** The distribution of AS events and the number of DAS genes in salt treated samples. **(B)** The top 20 GO terms enriched in the DAS genes. **(C)** Flow chart to analyze the distribution of the 10588 DEGs and 3709 DAS genes. **(D)** List of significant DAS genes detected in the study.

A gene was considered to incur a differentially alternative spliced (DAS) event when at least one of the AS transcripts was significantly expressed at a log2 fold change≥1 with adjusted *p* value < 0.05. When compared to the S0 group, a total of 2,169 DAS events in S1, 3,479 in S3, 3,092 in S6 and 2,669 in S12 were identified (**Figure 8A**; **Supplementary Tables S15**, **S16**). As some genes were alternatively spliced by more than one patterns, DAS events eventually generated a total of 3,709 DAS genes induced by salt stress, including 1164 DAS genes in S1, 1855 in S3, 1658 in S6 and 1429 in S12 groups (**Supplementary Tables S15**, **S17**). Although the highest counts of total raw AS events was observed in S6 group (**Figure 7B**), S3 group possessed the highest number of DAS events and genes, suggesting that more extensive changes occurred at 3 hours after salt treatment. While less than 10% DAS events were found to be differentially alternative spliced by RI pattern (**Supplementary Table S15**), SE was the most abundant DAS event that occurred under salt stress (**Figure 8A**; **Supplementary Table S15**).

GO enrichment analysis was performed on the DAS genes (**Figure 8B**; **Supplementary Table S18**). The top GO terms were mostly related to cellular anatomical entity, catalytic activity, membrane-bound organelle, nitrogen compound metabolic process and macromolecule metabolic process.

Venn diagram revealed that 368 DAS genes commonly alternatively spliced during the 12-hour salt treatment process (**Supplementary Figure S4A**; **Supplementary Table S19**). The top 20 GO terms enriched in the 368 common DAS genes were largely related to mitotic cycle, cytoskeleton and protein kinase (**Supplementary Figure S4B**; **Supplementary Table S20**).

### The combined analysis on DEGs and DAS genes in response to salt stress

Comparison between DEG and DAS gene datasets revealed that 2,002 genes were differentially expressed due to the changes of AS events, while 1707 genes exhibited DAS-only events (**Figure 8C**; **Supplementary Figure S5A**; **Supplementary Table S17**). When expanded at each time point, the number of the overlapped genes between DEG and DAS events were 512 in S1 vs S0, 803 in S3 vs S0, 895 in S6 vs S0 and 689 in S12 vs S0 pairwise groups (**Supplementary Figures S5B–E**).

Compared to the GO analysis on the all 3,079 DAS genes (**Figure 8B**), the 1,707 DAS-only genes exhibited similar pathway enrichment on cellular anatomical entity, catalytic activity, membrane-bound organelle, nitrogen compound metabolic process and macromolecule metabolic process (**Supplementary Figure S6A**; **Supplementary Table S18**). When focusing on the 2,002 common genes between DEGs and DAS genes, however, mitotic cycle relevant pathways, such as spindle assembly, chromatid segregation and nuclear division, were found to be enriched (**Supplementary Figure S6B**; **Supplementary Table S18**).

Further investigation on the DAS genes revealed 117 genes coding serine/threonine-protein kinase (**Figure 8D**; **Supplementary Table S17**). Other profound gene families were pentatricopeptide repeat (PPR)-containing protein (96 genes) and E3 ubiquitin ligase (76 genes) (**Figure 8D**; **Supplementary Table S17**). Some TF families like ZF, MYB and bHLH were also detected. Among these gene families, most of the PPR-coding genes were detected in DAS only group.

### Verification of AS patterns in DAS genes by RT-PCR

**Figure 9:**
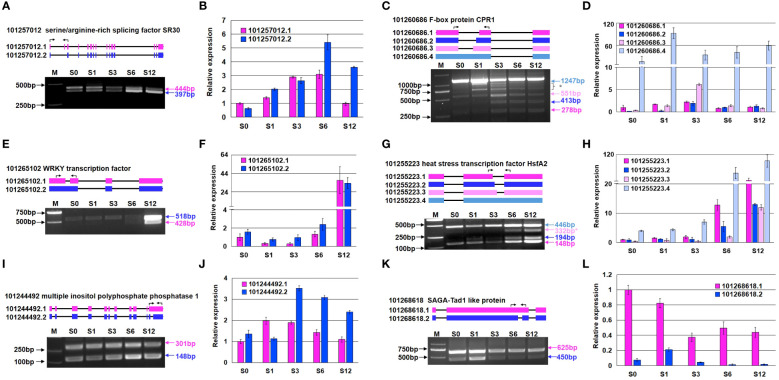
Validation of AS events in six representative genes by sqRT-PCR and qRT-PCR. **(A, B)** Expression of two transcripts of a serine/arginine-rich splicing factor SR30-like protein (101257012). **(C, D)** Expression of four transcripts of a F-box protein (101260686). **(E, F)** Expression of two transcripts of a WRKY transcription factor (101265102). **(G, H)** Expression of four transcripts of a heat stress transcription factor HsfA2 (101255223). **(I, J)** Expression of two transcripts of a multiple inositol polyphosphate phosphatase (101244492). **(K, L)** Expression of two transcripts of a SAGA-ted1-like protein (101268618). Panels **(A, C, E, G, I, K)** show the results of sqRT-PCR, and panels **(B, D, F, H, J, L)** depict the results of qRT-PCR. The asterisk (*) next to the band represents an unknown or abnormal alternative splice form. The black arrow on top of diagram indicates the location sites of the specific primers used for sqRT-PCR. Molecular markers are labeled on the left side, and the size of each transcript on the right side of gel picture. The transcript expression levels in panels **(B, D, F, H, J, L)** were relative to the transcript 1 of each gene and obtained from three independent replicates. The primers used for RT-PCR are listed in **Supplementary Table S22**.

Six genes were selected to validate the alternative splicing pattern under salt stress by RT-PCR (**Figure 9**). In the study, semi-quantitative RT(sqRT)-PCR was performed to visualize the patterns of splice isoforms based on size disparity between differentially spliced transcripts (Harvey and Cheng, 2016), and quantitative RT(qRT)-PCR were carried out to quantify the expression level of each transcript. While the relative expression level of most of transcripts studied by qRT-PCR were consistent with the RNA-seq results (**Figure 9**; **Supplementary Table S17**), we observed more complex splicing events in some genes based on sqRT-PCR. Salt stress apparently increased skipping frequency of the second exon in the gene coding for a serine/argnine-rich splicing factor SR30 (101257012) (**Figures 9A, B**). The actual splicing pattern of F-box protein CPR1 gene (101260686) was more complicated than expected (**Figures 9C, D**). Except for the increase of splicing at alternative 3’ splice site, extra bands were observed in the PCR products. A WRKY transcription factor (101265102) was highly induced at 12 hours after salt treatment, with an extra splicing variant detected at 6 and 12-hour treatment (**Figures 9E, F**). The increase of various splicing at alternative 5’ splice site contributed to the expression increase of a heat stress transcription factor HsfA2 (101255223) (**Figures 9G, H**). Intron retention caused the increased expression of a gene coding multiple inositol polyphosphate phosphatase (101244492) (**Figures 9I, J**). While the expression level of a gene coding for SAGA-Tad1 like protein (101268618) (**Figures 9K, L**) was not significantly changed, the composition of splicing variants altered due to intron retention.

### The potential DEG genes responsible for AS events under salt stress

**Figure 10:**
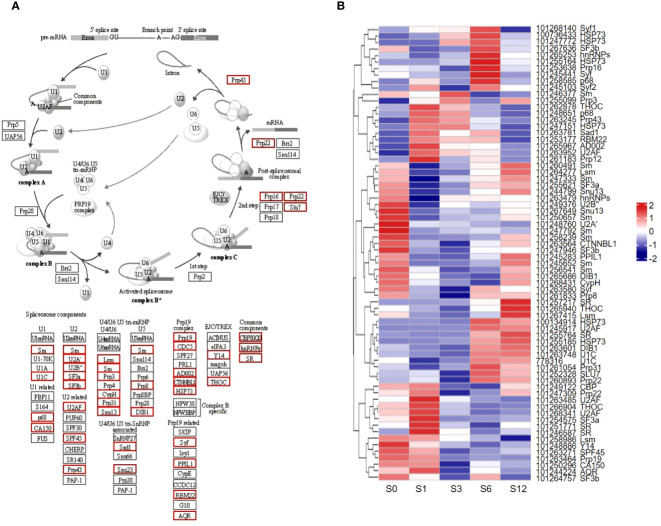
Differential expression of splicing related genes. **(A)** The spliceosome pathway based on KEGG analysis. Red boxes indicate differentially expressed genes. **(B)** Heatmap of differentially expressed spliceosome-related genes. NCBI gene IDs and potential gene names are listed on the right.

Given that the AS events were significantly altered in tomato root’s response to salt stress, analysis of potential genes responsible for AS events in response to salt stress was performed (**Figure 10**; **Supplementary Table S18**). Based on the spliceosome pathway obtained from KEGG database (**Figure 10A**), more than 100 genes were found differentially expressed in one or more salt treated samples (**Figure 10B**; **Supplementary Table S18**). These genes encoded 42 types of splicing relevant factors, such as SR splicing factors, Prp family proteins, SnRNP proteins and other factors in U1, U2, U5, U4/6 complexes. Their expression showed dynamic changes throughout the salt stress treatment (**Figure 10B**; **Supplementary Table S18**).

## Discussion

Salinity is one of the most significant environmental factors adversely affecting crop growth, development and yield. Tomato is moderately sensitive to salinity stress with seedlings especially susceptible due to its sensitive osmotic potential which is readily disrupted by salt stress during growth (Cuartero et al., 2006; Tanveer et al., 2019). Consequently, understanding of the underlying mechanisms of salinity tolerance will contribute to the breeding of salt tolerant tomato cultivars. To explore the gene regulatory network of tomato to salt stress, we investigated the early transcriptional responses to salt treatment over a 12-hour period in tomato roots.

The early morphological changes by tomato to salt stress (**Figure 1**) is consistent with the view that salt-specific signaling pathways are rapidly triggered in plant roots during the very early stages of salt stress (Galvan-Ampudia et al., 2013; Choi et al., 2014). The response of plant roots to salt stress involves complex regulation of gene expression at multiple levels, including at transcription, post-transcription, translation, post-translation, and metabolism, which eventually result in phenotypic changes (Barkla et al., 2013; van Zelm et al., 2020). Here we show that transcriptomic analysis of tomato roots under salt stress revealed a considerable and dynamic expression of transcripts in tomato roots during the early 12-hour treatment process of salt exposure. The gene expression patterns of continuously up or down-regulation, peak expression at 1 or 3 hours, and reduction at the first 3 hours were highly pronounced (**Figure 2**). The GO terms enriched in those clusters revealed the important changes of key biological processes, such as hormone signaling, cell cycle, amino acid metabolism and response to oxidative stress, during the 12-hour salt treatment.

This study revealed that amino acid metabolism was greatly enhanced at the early response of tomato root to salt treatment (**Figure 2**; **Supplementary Table S7**; **Supplementary Figure S1**). Amino acid metabolism is involved in various strategies during plant adaption to abiotic stress conditions (Huang and Jander, 2017; Hildebrandt, 2018; Batista-Silva et al., 2019; Reshi et al., 2023). Accumulation of free amino acids have been generally observed in diverse plants under various abiotic stress (Hildebrandt et al., 2015; Huang and Jander, 2017) and the enhancement of amino acid biosynthesis and amino acid transmembrane transport have been reported to improve plant tolerance to salt stress (Batista-Silva et al., 2019; Shohan et al., 2019). While some amino acids like proline are known to be potential ROS scavengers to protect plant cell from oxidative damage (Hayat et al., 2012), several amino acids, such as phenylalanine, tyrosine and tryptophan, arginine, methionine and lysine, act as precursors for the synthesis of nitrogenous secondary metabolites and signaling molecules (Tzin and Galili, 2010; Batista-Silva et al., 2019; Heinemann and Hildebrandt, 2021). Therefore, enhancement of amino acid metabolism is likely to be an important adaptive strategies to eliminate the adverse effects of salt stress in tomato root. On the other hand, it’s known that the high levels of ROS concentration can affect amino acid metabolism, specially the site-specific chemical modification of amino acids such as arginine, lysine, proline, threonine and tryptophan, which cause increased vulnerability to proteolytic degradation (Moller et al., 2007). In this study, most of the DEGs involved in response to ROS and oxidative stress exhibited a continuously up-regulated expression pattern, suggesting the continuous accumulation of ROS throughout treatment of salt stress (**Figure 2**; **Supplementary Table S7**, cluster 9). Thus, the ROS accumulation induced by salt stress may contribute to the considerable changes of amino acid metabolic and catabolic processes under salt tress.

Plant hormones play vital roles in maintaining plant growth and enable plants to survive under conditions of salt stress (Ryu and Cho, 2015; Yu et al., 2020). It was reported that tomatoes could adapt to salt stress by dynamically regulating their hormone levels to establish new hormone balance (Wang et al., 2023a). The levels of ABA, SA, and JA and their respective signal transduction pathways were reported to be significantly increased, while decrease in the levels of GA and IAA were observed during the early response to salt stress (Wang et al., 2023a). In the current study, we also observed the dynamic regulation of plant hormone signaling transduction. Regulation of ABA and auxin-mediated signaling pathways were found to be significantly pronounced throughout the early response to salt stress (**Figure 5**; **Supplementary Tables S7**, **S11**). ABA is the primary hormone that promotes plant salt tolerance (Sah et al., 2016; Vishwakarma et al., 2017; Pye et al., 2018) where auxins promote plant growth (Zhang et al., 2022). The majority of genes involved in both hormone pathways appeared in expression clusters exhibiting continuous down-regulation or enhanced expression within the first 6 hours. This suggests that the regulation of salt tolerance and growth are closely intertwined. In contrast to a previous study (Wang et al., 2023a), significant regulation of the SA-mediated signaling pathway were not detected. On the other hand, response to JA was found to be prominent throughout the early response to salt stress (**Supplementary Table S7**). A previous study by Abouelsaad and Renault (2018) found that activation of JA signaling pathway enhanced tomato salt tolerance, aligning with our current result.

Cytokinin is another important hormone that modulates plant development and tolerance to various environmental stimuli (Mandal et al., 2022; Papon and Courdavault, 2022; Yin et al., 2023) by regulating cell cycle and differentiation, promoting antioxidant systems, impeding plant senescence, and cross-talking with stress-related phytohormones (Liu et al., 2020; Mandal et al., 2022). While defective cytokinin signaling mitigates high salinity in *Arabidopsis* via regulation of the lipid and flavonoid gene-to-metabolite networks, enhancement of cytokinin content was reported to improve tomato salt tolerance in tomato (Zizkova et al., 2015). We noticed that the pathway of response to cytokinin was only enriched in the cluster of down-regulated DEGs (**Figure 2**, cluster 10; **Supplementary Table S7**), suggesting that cytokinin-mediated signaling pathway was suppressed during the early response of tomato root to salt stress.

Transcription factors (TFs) play a central role to regulate the expression of the genes responsible for plant stress tolerance. Numerous TFs from the families like bZIP, NAC, WRKY, MADS, MYB, ZF, HSF and bHLH families are involved in conferring salt tolerance in various crop species (Duan et al., 2019; Zang et al., 2019; Li et al., 2020; Guo et al., 2021; Li et al., 2021; Wang et al., 2021a, Wang et al., 2021b; Liu et al., 2023; Rosca et al., 2023; Sukumaran et al., 2023; Ye et al., 2023; Wang et al., 2023b), including in tomato (Pan et al., 2010, Pan et al., 2012; Wang et al., 2013; Klay et al., 2014; Campos et al., 2016; Bai et al., 2018; Klay et al., 2018; Li et al., 2018; Waseem et al., 2019; Zhang et al., 2020; Guo et al., 2021; Qian et al., 2021; Chen et al., 2022) In this study, more than 700 TFs were found to be differentially expressed in tomato roots under salt stress (**Supplementary Table S13**) with members from MYB, ZF, bHLH and AP2/ERF gene families being the most abundant. Among these salt responsive TFs, several of them were previously reported to modulate tomato salt tolerance. For example, a R1-MYB type TF coding gene, *SlARS1* (Gene ID 101257705), that was reported to affect ABA-mediated stomatal conductance under salt stress (Campos et al., 2016), was found to be significantly induced under salt stress especially at 3-hour salt induction (**Supplementary Table S13**); SlWRKY13 previously proved to be negative regulator of tomato salt tolerance (Birhanu et al., 2020) was among the decreased WRKY TF group in this study. We also observed significant down-regulation of several AP2/ERF family TFs (**Supplementary Table S13**), such as *SlERF.B1* (Gene ID 543867) (Wang Y. et al., 2022) and *SlERF.B3* (Gene ID 108511945) (Klay et al., 2014) that negatively regulate tomato salt tolerance. Given the key roles of transcription factors in regulation of salt response in plants, the salt-sensitive TFs identified in this study deserve further investigation in the future.

Alternative splicing (AS) is an important post-transcriptional mechanism that regulates plant growth and development and is prevalent during stress (Kelemen et al., 2013; Jabre et al., 2019; Punzo et al., 2020; Rosenkranz et al., 2022). In tomato, around 65% of the annotated protein-coding genes possess multiple transcript isoforms (Clark et al., 2019). Alternative splicing changes have been reported in tomato plants grown under phytotron vs greenhouse conditions (Wang et al., 2017), in inflorescences of cultivated and wild tomato species (Zhou et al., 2022), during fruit development regulation (Sun and Xiao, 2015; Wang et al., 2016), pollen responses to heat stress (Keller et al., 2017), tomato responses to drought stress (Lee et al., 2020), water deficit stress (Ruggiero et al., 2022), low nitrate stress (Ruggiero et al., 2022), phosphate starvation (Tian et al., 2021), and response to the fungal infection by *Trichoderma harzianum* (De Palma et al., 2019). Based on these studies, differential alternative splicing (DAS) was found to be tissue-specific, developmental stage-related or stress-responsive condition. As there is limited understanding regarding the involvement of alternative splicing in tomato’s response to salt stress, we investigated the changes of AS events during early response of tomato root to salt stress in this stduy (**Figure 3A**, **4B**). A total of 46,115 AS events, including A5SS, A3SS, RI, MXE and SE, were detected in the tomato root transcriptome (**Figure 7**; **Supplementary Table S14**), revealing a comprehensive and dynamic alteration in AS patterns in tomato roots during early responses to salt stress. An integrated genome-wide study (Clark et al., 2019) reported that RI was the prevalent AS event (18.9%) followed by alternative A5SS and A3SS, while SE was the least AS type, accounting for only 6%, among total 369,911 AS events in tomato. By contrast, SE was the most abundant AS event in tomato root under salt stress (**Figure 7**; **Supplementary Table S14**), suggesting that the alternative splicing pattern of SE might be susceptible to salt stress in tomato root. The dominance of SE in AS events was also previously reported in tomato root and shoot during phosphate starvation (Tian et al., 2021) and in date palm seedlings under salt stress (Xu et al., 2021), suggesting that AS patterns are not constant, but may change depending on the abiotic condition. On the other hand, RI event was reported to be the most frequent event induced by salt stress in *Arabidiopsis* (Ding et al., 2014b), wheat (Liu et al., 2018), cotton (Zhu et al., 2018) and Barley (Fu et al., 2019), while A3SS was the mostly affected AS events in rice by salt stress (Fu et al., 2019). Given that the differences in AS profiles are related to tissue type, stress condition and genotype (Gan et al., 2011; Vitulo et al., 2014; Martín et al., 2021; Zhou et al., 2022), the differences on the alternative splicing preference induced by salt may contribute to the evolutionary adaptation process in tomato.

Salt-induced AS of non-differentially expressed genes may contribute to the transcriptome reprogramming for salt tolerance of tomato root. Interestingly, except for unclassified genes, differentially alternative splicing induced by salt stress in tomato root were largely detected in the gene families of serine/threonine-protein kinase, PPR-containing protein, and E3 ubiquitin ligase (**Figure 8D**). Serine/threonine-protein kinases are key enzymes that reversibly phosphorylate the OH group of serine or threonine residues at the post-translational level. The network of serine/threonine kinases in plant cells is considered a central unit to accept and convert signaling information from sensing receptors of various stimulus and phytohormones and in turn guide responsive changes in gene expression, metabolism, plant growth and development (Hardie, 1999). One of the most representative serine/threonine-protein kinases belong to the SnRK2 family, which are involved in the ABA-dependent signaling pathway to regulate plant development and plant responses to diverse abiotic stresses (Kulik et al., 2011). Ubiquitin E3 ligases are major players that catalyze the covalent attachment of ubiquitin to target proteins (Mazzucotelli et al., 2006; Kelley, 2018). Ubiquitination of substrates is a dynamically regulated process and can generate diverse functional outcomes like potential degradation or activation of target proteins and changes in subcellular localization (Kelley, 2018). E3 ubiquitin ligases are thus well-known to be central regulators of many plants molecular processes, including plant hormone biosynthesis, signaling transduction and response to various stress conditions (Wang S. et al., 2022). AS susceptibility of serine/threonine kinases and E3 ubiquitin ligases in tomato root under salt stress poses as an additional complication in understanding the relationship between hormone signaling transduction and salt-responsive gene regulation. Pentatricopeptide (PPR) proteins are characterized by tandem arrays of a degenerate 35-amino-acid sequence motifs (Lurin et al., 2004). They are a large family of modular RNA-binding proteins with essential roles in organelle biogenesis, RNA editing, mRNA maturation and thus involved in many diverse biological processes during plant growth, development and stress acclimation (Barkan and Small, 2014). A previous genome-wide analysis revealed that the tomato genome has 471 PPR-coding genes (Ding et al., 2014a). In this study, extensive AS occurred in PPR-coding genes under salt stress as 96 out of the 471 PPR-coding genes were found to be differentially alternative spliced (**Figure 8D**; **Supplementary Table S17**). The dynamic AS changes of PPR-coding genes may also contribute to the gene regulation and transcriptome reprogramming under salt stress.

We also observed that some genes, such as the genes coding CPR1-like F-box protein and HsfA2 in **Figure 9**, were abnormally spliced under salt. These observations indicate that AS modulation in response to salt stress is more complicated than previously envisioned and that modulation of alternative splicing deserves more attention in future studies. The genes and their transcripts identified in the present study can be targeted for the improvement of tomato salt tolerance.

Analysis on the differential expression of spliceosome pathway-associated proteins (**Figure 10**) revealed the potential roles of specific groups of AS-associated proteins in regulating tomato root response to salt stress. Expression of many genes coding key component assembled in spliceosome machinery, such as small nuclear ribonucleoprotein complexes (snRNPs), U1, U2, U4, U5, and U6, was found to be affected in tomato root under salt stress. Except for core components of spliceosome machinery, the expression of trans-factors, including serine/arginine-rich (SR) proteins and heterogeneous nuclear ribonucleoprotein (hnRNP), were also significantly regulated under salt stress (**Figure 10**; **Supplementary Table S21**). In addition to differential expression, alternative splicing in some of AS-related genes, such as SR-like splicing factors (**Figure 8D**; **Supplementary Table S17**), was also observed, which adds an additional complication of AS regulation during salt stress. It is reported that the salt-responsive regulation of SR gene isoforms may result in inaccurate identification of splicing sites and destabilization of the spliceosome complex (Albaqami et al., 2019; Xu et al., 2021; Laloum et al., 2023). Therefore, future studies on the AS events of splicing-related proteins will provide new insights on how genes are regulated in salt-stressed tomato.

Collectively, this study provides a comprehensive view of transcriptome changes and highlights the key role of AS in tomato root response to salt stress. A large number of DEGs and DAS genes involved in diverse metabolic pathways, such as hormone signaling transduction, DNA transcription, RNA binding and processing, were identified. The findings in this study expand our current understanding of transcriptional and post-transcriptional regulation in the response of tomato roots to salinity stress and provide an important gene resource for developing salt-tolerant tomato plants.

## Materials and methods

### Plant materials and salt stress treatment

Tomato seeds (*S. lycopersicum* cv. Ailsa Craig) were sown in a nutrient soil mixture with a ratio of 3:1 (w/w) and cultivated in an illumination incubator under standard conditions (16 hours of light at 26°C, followed by 8 hours of darkness at 20°C). After three weeks, the seedlings were transferred to pots filled with 1/2 Hoagland’s nutrient solution following root rinsing under running water. After two additional weeks, seedlings of uniformed size were selected and treated with 150 mM NaCl. Three biological replicates of root samples were collected at 0, 1, 3, 6, and 12 hours post treatment. All samples were immediately frozen in liquid nitrogen and stored at -80°C for further use.

### RNA extraction, library construction and sequencing

Total RNAs were extracted from root samples with TransZol UP Plus RNA kit (Tiangen Biotech, China). The mRNAs used for cDNA library construction were isolated from total RNAs using oligo-dT magnetic beads. A total of 15 cDNA sequencing libraries were constructed and sequenced using the DNBSEQ™ technology (Beijing Genomics institution, China) following the manufacturer’s recommendations to generate paired-end sequencing data.

### RNA-seq analysis

The raw sequencing data was filtered by SOAPnuke v1.5.6 (https://github.com/BGI-flexlab/SOAPnuke) to remove low-quality reads, and the high-quality reads were mapped against the reference *S. lycopersicum* genome (Version SL3.1, https://www.ncbi.nlm.nih.gov/datasets/genome/GCF_000188115.5/) using the HISAT2 software (v2.1.0) (http://www.ccb.jhu.edu/software/hisat/index.shtml) with default parameters. Detection of differentially expressed genes was performed using Bowtie2 (v2.3.4.3) (http://bowtiebio.sourceforge.net/Bowtie2/index.shtml) and DESeq2 (v1.4.5) (http://www.bioconductor.org/packages/release/bioc/html/) with default parameters. The mapped reads were counted and normalized into fragments per kilobase of transcript per million (FPKM), and the expressed genes with a log2 fold change≥1 and adjusted *p* value < 0.05 were identified as differentially expressed genes (DEGs). Gene clustering was analyzed using the software of Dynamic Trend Analysis on https://www.omicshare.com/tools. Significantly enriched trends were determined according to a significance threshold *p* value<0.05 (Ernst and Bar-Joseph, 2006). Alternative splicing analysis were performed using rMATS (V3.2.5) (http://rnaseq-mats.sourceforge.net) with default parameters. Compared to control samples, alternative splicing events with adjusted *p* value < 0.05 were identified as differentially alternative spliced (DAS) events, and the genes that had at least one of the transcripts differentially expressed (log2 fold change≥1 and adjusted *p* value < 0.05) were considered to be DAS genes.

### Pathway enrichment analysis

Gene Ontology (GO) analysis of the candidate gene groups was performed on https://geneontology.org/ that is powered by PATHER. Annotation version used in GO enrichment was GO Ontology database DOI:10.5281/zenodo.10536401 released on Jan 17, 2024. Kyoto Encyclopedia of Genes and Genomics (KEGG) enrichment was performed using KEGG Mapper on https://www.genome.jp/kegg/. The GO terms and KEGG pathways with *p* value<0.05 were defined as significantly enriched in the candidate gene groups.

### Validation of alternative splicing

Semi-quantitative RT(sqRT)-PCR and quantitative RT(qRT)-PCR were performed to verify the AS pattern of six representive genes. Total RNA was subjected to first-strand cDNA synthesis using *EVo M-MLVRT* Mix Kit with gDNA Clean for qPCR Ver.2 (Vazyme, Nanjing, China) following the manufacturer’s instructions. Specific primers of target genes (**Supplementary Table S22**) were designed using the NCBI primer design tool (https://www.ncbi.nlm.nih.gov/tools/primerblast). sqRT-PCR was conducted with HotStarTaq Plus DNA Polymerase Reagents (Qiagen) and the melting temperature (Tm) was optimized based on different sequences of the primers. PCR products were visualized via horizontal gel electrophoresis using a 2% agarose-TBE gel. Reactions of qRT-PCR were carried out on Applied Biosystems StepOnePlus instrument using SYBR Green Premix *Pro Taq* HS qPCR Kit (Vazyme, Nanjing, China). The gene encoding ribosomal protein L2 (RPL2) (Løvdal and Lillo, 2009) was used as internal reference for qRT-PCR. Three independent replicates were tested for each transcript.

## Data availability statement

The sequencing data are available at https://www.ncbi.nlm.nih.gov/sra/ at NCBI with accession number PRJNA1110838.

## Author contributions

JG: Data curation, Investigation, Writing – original draft. YQ: Data curation, Formal analysis, Writing – original draft. YT: Investigation, Writing – original draft. LZ: Data curation, Writing – review & editing. TWO: Validation, Writing – review & editing. YY: Conceptualization, Methodology, Writing – original draft. LT: Conceptualization, Data curation, Supervision, Writing – original draft, Writing – review & editing.
