# Supplementary material for: RNA-seq analysis reveals transcriptome reprogramming and alternative splicing during early response to salt stress in tomato root
Source: Front Plant Sci. 2024 Jun 20;15:1394223. doi: 10.3389/fpls.2024.1394223 (PMC11222332; doi:10.3389/fpls.2024.1394223)
Supplement: Supplementary file 2 [file Image_1.pdf]

Supplemental data

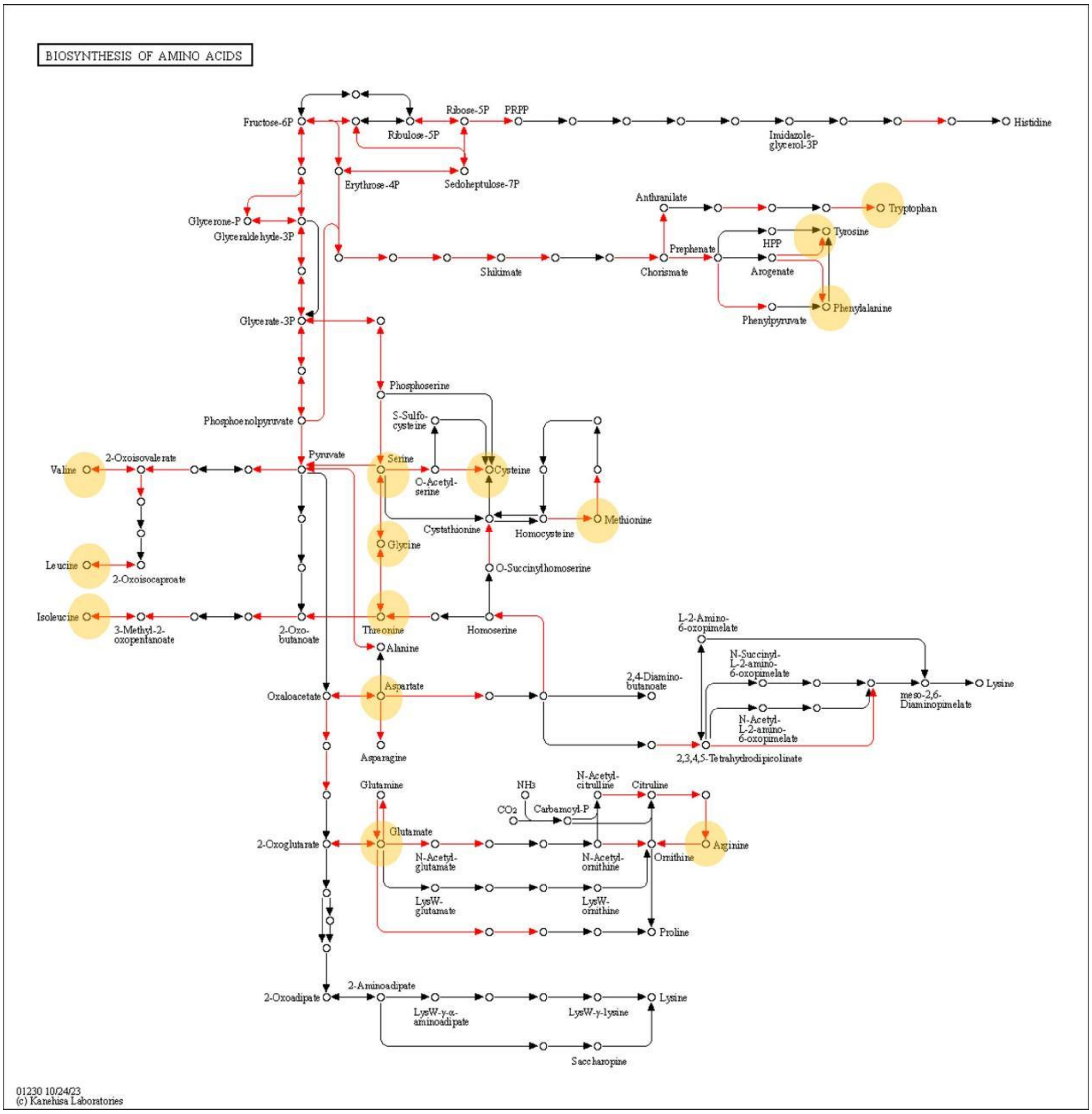

Figure S1

Overview of amino acid biosynthesis pathway via KEGG analysis. The annotated DEGs in RNA-seq analysis were mapped against KEGG pathway maps (www.kegg.jp/kegg). Red arrows and yellow dots were used indicate to label the steps and amino acids significantly influenced by salt treatment.

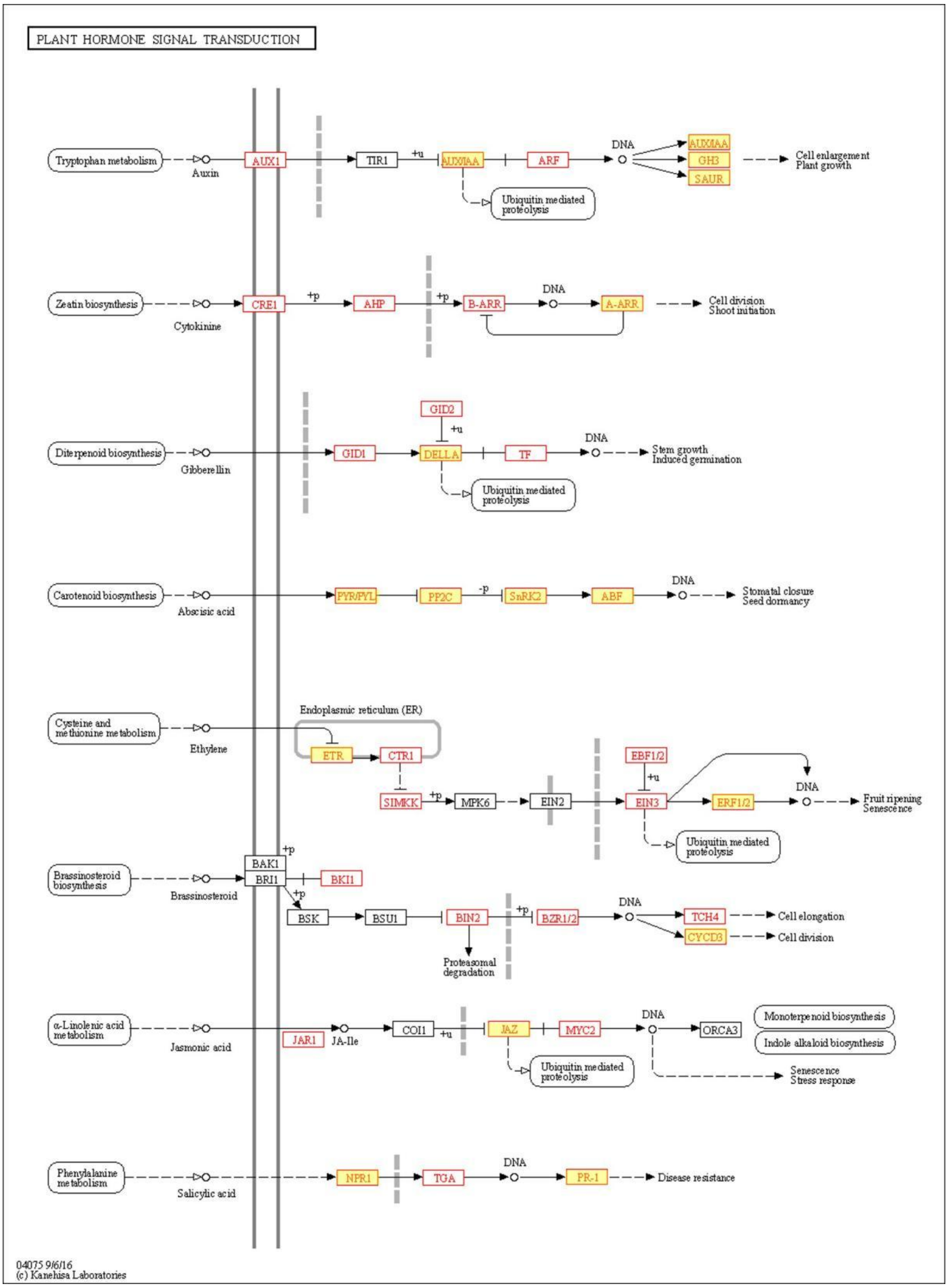

Figure S2

Overview of plant hormone signaling transduction pathway via KEGG analysis. Red boxes indicated the key factors significantly enriched in DEGs. The boxes highlighted in yellow were used to label the key factors presented in the common DEGs among salt-treated samples.

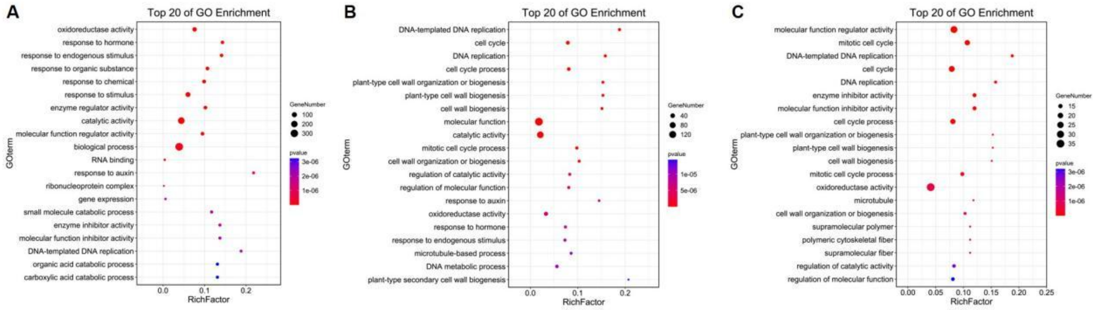

Figure S3

Top 20 GO terms enriched in the common genes among salt-treated groups. (A) The top 20 GO enrichment terms of all the common DEGs among salt-treated groups. (B) The top 20 GO enrichment terms of the common up-regulated DEGs. (C) The top 20 GO enrichment terms of the common down-regulated DEGs.

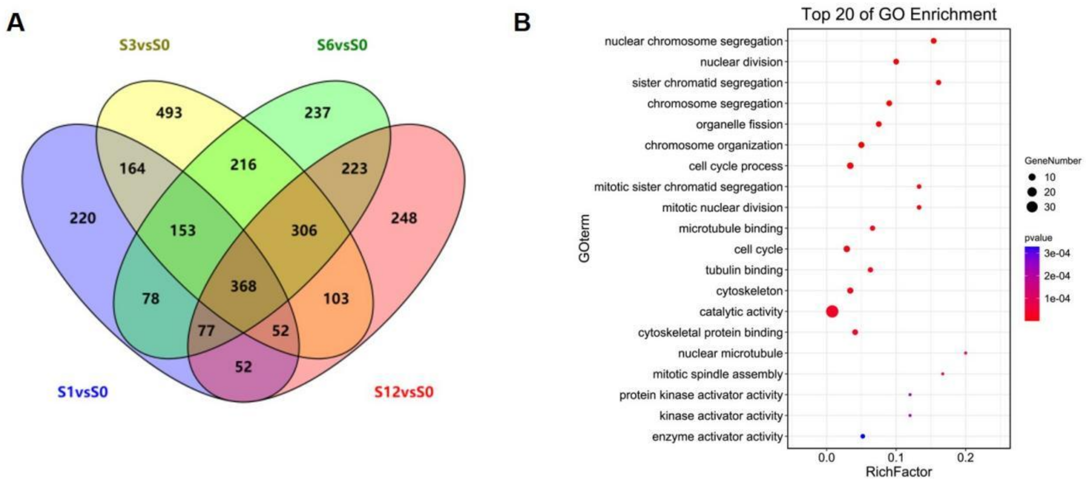

Figure S4

The overlapping DAS genes at different time points after salt treatment. (A) Venn diagram representation of the overlapping genes commonly presented in the DAS genes of all salt-treated groups. (B) The top 20 GO terms enriched in the common 368 DAS genes.

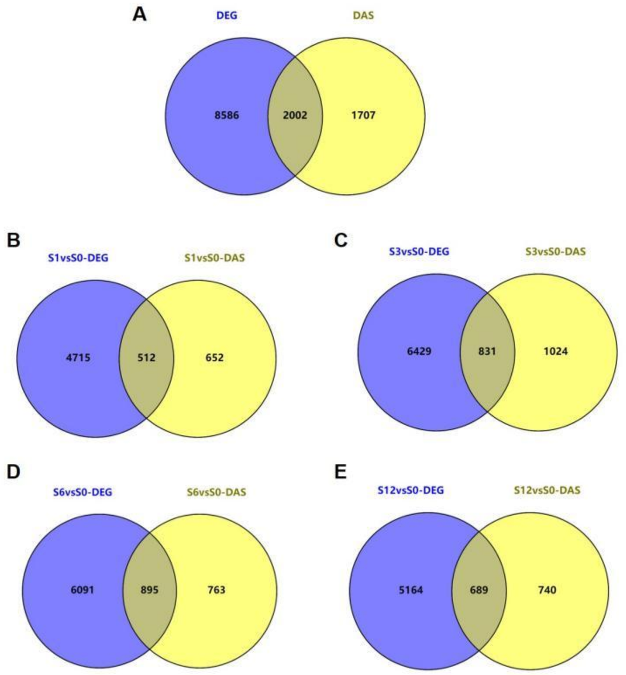

Figure S5

Venn diagram analysis on the overlap of DEGs and DAS genes at different time points after salt treatment. (A) Overlap of total DEGs and DAS genes. (B-E) Overlap of the DEGs and DAS genes identified at 1 (B), 3 (C), 6(D) and 12 (E)-hour salt treatment.

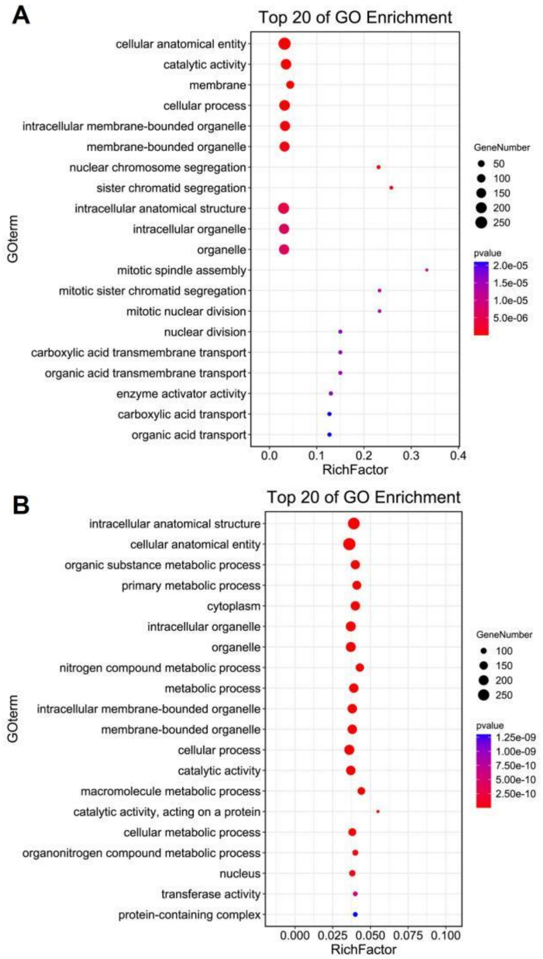

Figure S6

Top 20 GO terms enriched in the up- (A) and down-regulated (B) DAS genes.
